# Light Sensitive Bumblebee Species Are Associated With Forest Habitat and Forest‐Dominated Landscapes

**DOI:** 10.1002/ece3.72351

**Published:** 2025-10-22

**Authors:** Océane Bartholomée, Pierre Tichit, Jens Åström, Henrik G. Smith, Sandra Åström, Markus A. K. Sydenham, Emily Baird

**Affiliations:** ^1^ Centre for Environmental and Climate Science Lund University Lund Sweden; ^2^ Department of Zoology Stockholm University Stockholm Sweden; ^3^ Department of Biology Lund University Lund Sweden; ^4^ Norwegian Institute for Nature Research Trondheim Norway; ^5^ Norwegian Institute for Nature Research Oslo Norway

**Keywords:** bumblebees, forest, light sensitivity, plant shade tolerance, species distribution, visual traits

## Abstract

We investigate whether the eye parameter of bumblebees—a visual trait measuring the tradeoff between light sensitivity and visual resolution—is associated with: (i) local habitats, (ii) forest cover at the landscape scale (1 km radius), and (iii) the shade tolerance of the plants they forage on. The association of bumblebee species with local habitat and forest cover at the landscape scale was analyzed using generalized linear mixed models. We combined data from the Norwegian national bumblebee monitoring program with Corine CLC+ land cover and bumblebee functional traits: eye parameter and intertegular distance. These analyses were done at the species and community level. To determine whether bumblebee light sensitivity correlated with the shade tolerance of the plant they forage on, we combined bumblebee–plant interactions from a British database with a Swedish plant trait database. Our findings showed that bumblebee species with high light sensitivity were more common and abundant in forest habitats and areas with greater forest cover, while species with high visual resolution showed the opposite trend. This pattern was reflected at the community level, as indicated by the community‐weighted mean of the eye parameter, which increased with forest cover and was higher in forest habitats. Furthermore, bumblebees with higher light sensitivity tended to forage on plants with greater shade tolerance. These results suggest that visual adaptations for light sensitivity contribute to shaping bumblebee species distributions across different scales. Our study underscores the importance of pollinator vision in understanding species niches and its value for species distribution modeling. Moreover, by relating pollinator visual abilities to plant niches for the first time, this study provides an important basis for future modeling of plant–pollinator interactions and targeted conservation measures for plants and pollinators in forested landscapes.

## Graphical abstract

Bumblebee species and bumblebee communities with high light sensitivity are more common and abundant in forest habitats and forested landscapes. Bumblebees with higher light sensitivity tend to forage on plants with greater shade tolerance. Our results suggest that visual adaptations for light sensitivity contribute to shaping bumblebee species distributions across different scales.

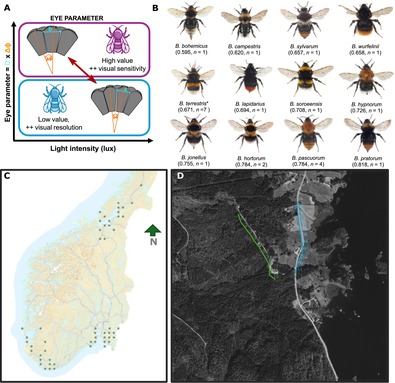

## 1 Introduction

Pollinator declines threaten ecosystem functioning, as animal pollination is crucial for the reproductive success of most flowering plants (Ollerton et al. 2011). A major driver of recent pollinator declines, including bees, is human‐driven changes in habitat availability and quality, resulting in loss of foraging resources and nesting sites (Potts et al. 2016; Potts et al. 2010). To enhance our ability to predict pollinator responses to environmental changes, it is essential to understand how habitat characteristics, such as temperature and light conditions, influence pollinator species distributions (Williams et al. 2010), with the potential to support conservation management actions (Porfirio et al. 2014; Villero et al. 2017). Species sharing ecological traits that affect their fitness can be expected to respond similarly (McGill et al. 2006), resulting in species sorting along environmental gradients through “trait‐based environmental filtering” (Vellend and Agrawal 2010).

Trait‐based approaches are increasingly used to describe and predict species responses to global changes using functional traits (Green et al. 2022), aiming to identify mechanistic links between community dynamics and environmental conditions (McGill et al. 2006). While species‐level trait values are commonly used in this context (e.g., Pradervand et al. 2014; Torvanger et al. 2025; Williams et al. 2010), recent studies highlight the importance of intraspecific trait variability and its role in biotic interactions (Arroyo‐Correa et al. 2023; Cantwell‐Jones et al. 2024). The traits of insect pollinators, such as bees, can be broadly classified into autecological traits describing the life histories of species, locomotive ability traits, and climatic tolerance traits (Martinet et al. 2021). A fourth category includes sensory traits that describe how pollinators respond to the sensory cues necessary for finding plants (Spaethe et al. 2001) and interacting with their environment (Bartholomée et al. 2023). For insect pollinators, body size (Green et al. 2022) is widely used as a functional trait, complicating mechanistic interpretation as it is correlated with several functions. The body size of bees, when used as a proxy for thermoregulatory abilities, relates to altitudinal gradients (Osorio‐Canadas et al. 2022; Peters et al. 2016) and, when used as a proxy for mobility, mediates responses to habitat fragmentation (Carrié et al. 2017; Gérard et al. 2021). Therefore, it is challenging to relate changes in mean body size within a community to a specific ecological mechanism. Analyses of trait–environment relationships have taught us how environmental conditions filter traits related to mobility and resource use (Hoiss et al. 2012; Sydenham et al. 2015). However, we lack a clear understanding of how insect pollinator species differ in their ability to navigate and forage efficiently under different light microhabitat conditions, as being active under a forest canopy in dim light can be visually challenging (Arnold and Chittka 2012).

**FIGURE 1:**
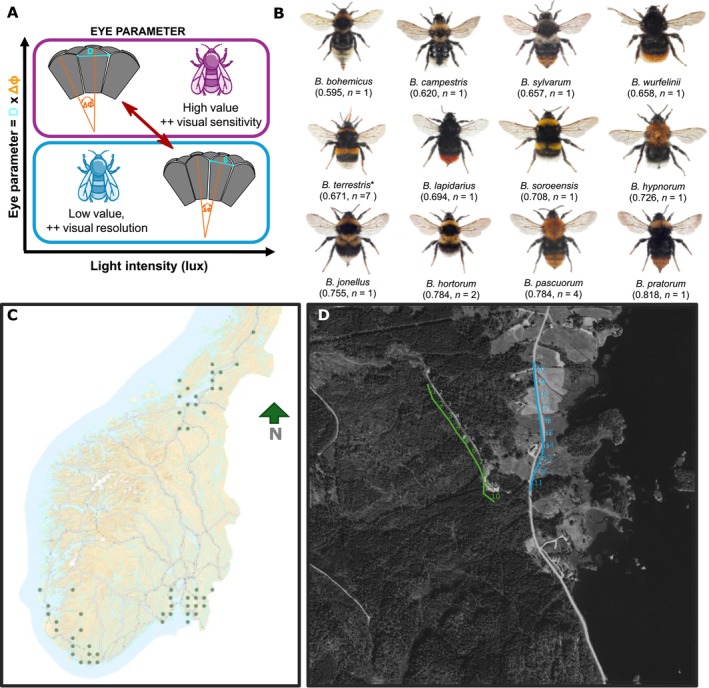
(A) Eye parameter reflects the trade‐off between spatial resolution and light sensitivity in insect compound eyes. To cope with reduced light intensity (top left), insects living in dim habitats typically have higher facet diameters (D, μm) and higher angle between each unit (Δ*φ*, rad) than their bright light counterparts (bottom right). (Adapted from Bartholomée et al. (2023)). (B) 11 bumblebee species from the Norwegian national inventory included in the analysis of Question 1 (pictures not on scale). Species are sorted by increasing values of eye parameter. Each picture is annotated with the species name (B. is for Bombus) (CC BY 3.0, Arnstein Staverløkk). In between bracket: Species‐specific eye parameter value (μm.rad) and sample size of the measurements (*n*). * *B. terrestris* includes here both *B. lucorum* and *B. terrestris* , as both species are highly difficult to distinguish in the field (Carolan et al. 2012). (C) Map of the monitored sites in Norway. (D) Close‐up of survey square 803 in the South‐Western region, with numbered open forest transects in green and grassland transects in yellow.

Sensory traits dictate how animals experience and interact with their environment (Dominoni et al. 2020; Elmer et al. 2021). Vision is essential for highly mobile animals like flying insects, as it helps them orient, find flowers (Spaethe et al. 2001), and commute between nests and food resources, especially for central place foragers like bees (Warrant et al. 2004). Recent studies have related eye size to light microhabitat use for damselflies (Scales and Butler 2016) and butterflies (Wainwright et al. 2023), but not in bumblebees (Bartholomée et al. 2023). Variations in a visual trait known as the eye parameter, which measures how an insect's compound eye balances light sensitivity (important in dim light conditions) and visual resolution (possible under bright conditions) (Jander and Jander 2002) (Figure 1A), explain how different bumblebee species use light microhabitats in hemi‐boreal forest understory at the scale of a light patch on the understory floor (i.e., the scale at which these pollinators forage) (Bartholomée et al. 2023). How sensory traits are associated with variation in pollinator communities at larger scales and across wider geographical ranges remains unclear. A recent study found that bumblebee communities in grasslands embedded in landscapes with a higher forest cover (within 1 km of the focal grassland) had higher eye parameter values, suggesting enhanced visual abilities under the dimmer light conditions of forests (Tichit et al. 2024). However, a more in‐depth investigation is needed to determine if this pattern is reflected across scales, both locally as differences between grassland and forest habitats and at landscape scales when the relative proportion of habitat types varies. While such analyses can indicate whether bumblebee species can navigate contrasting habitats, they do not provide information on their ability to forage in these varied light habitats, as vision is involved in both microhabitat use (Bartholomée et al. 2023) and flower detection (Spaethe et al. 2001). However, this can be investigated by testing if the niche of plant communities foraged by bumblebees is consistent with their visual abilities, thereby strengthening the visually mediated community assembly.

Here, we test whether forests are associated with bumblebee species that have higher light sensitivity, which we hypothesize increases their relative fitness in shaded microhabitats. We do this at both local and landscape scales by contrasting forest and grassland habitats and comparing landscapes with varying forest cover percentages. We used data on bumblebee species distributions from long‐term structured surveys conducted in both grasslands and open forests (Åström and Åström 2023). Due to the complexity and time required to obtain this calculation, the eye parameter trait was measured on a limited number of bumblebee individuals sampled outside the study monitoring range (Taylor et al. 2019; Tichit et al. 2024). However, it showed that their visual traits were adequate for flying in such habitats. To investigate if their visual abilities also enable them to locate and land on flowers—a task requiring more complex visual processing—we examined whether bumblebees with higher light sensitivity exhibit a stronger preference for shade‐tolerant plants compared to those with higher visual resolution. Such a finding would suggest that these species can both navigate and forage effectively in low‐light environments. To understand the interplay between bumblebee vision and the light niche they forage in, we focused on plant‐bumblebee interactions extracted from an open‐source database (Database of Pollinator Interactions) (Balfour et al. 2022) combined with a trait database gathered for Swedish plant species (Tyler et al. 2021).

## 2 Material and Methods

### 2.1 Bumblebee Monitoring Data

Since 2009, the Norwegian Institute for Nature Research (NINA) has managed a nationwide bumblebee monitoring program in both open lowland (below the treeline, referred to as grasslands) and forests, with data available on GBIF (Åström and Åström 2023). Our focus is on 9 years of sampling from 2013 to 2021, covering three geographical regions (58.06°–64.93° N, 5.22°–13.18° W, Figure 1C). Monitoring was conducted at 52 sites at low altitudes (mean [min—max]: grasslands: 110 [0–487] m.a.s.l; forests: 195 [3–722] m.a.s.l.), each site covering 1.5 × 1.5 km squares spaced at least 18 km apart. At each site, a 1 km transect (20 × 50 m segments) was walked three times per year. Transects within each region were equally divided between open forest and grassland habitats (Figure 1D). First observations were carried out within the leaf‐out periods of some tree species while the second and third were conducted after leaf‐out. The transect walks in forests and open habitats were conducted simultaneously, ensuring a relevant relative difference in light conditions between both habitat types. Monitoring was carried out between 10:00 and 17:00 under suitable weather conditions for insect activity (> 15°C, < 60% cloud cover, weak wind). Bumblebees were counted and identified visually within 2.5 m on each side of the transect and 5 m ahead of the transect. Our analysis focused on the 12 most abundant species (≥ 30 individuals observed over 9 years), including 10 noncuckoo species and two cuckoo species ( *B. bohemicus* and *B. campestris* ) (Figure 1B), resulting in a dataset of 47 sites. Despite differing life history traits, both cuckoo and noncuckoo species were included as they possess sensory traits enabling them to be active in the habitats they are observed (Table S1). The species reported as *Bombus* sensus stricto was interpreted as the *B. terrestris* complex, which can include *B. terrestris* , *B. lucorum* , *B. magnus* , and *B. cryptarum*, which are difficult to separate in the field (Carolan et al. 2012).

### 2.2 Landscape Composition

**FIGURE 2:**
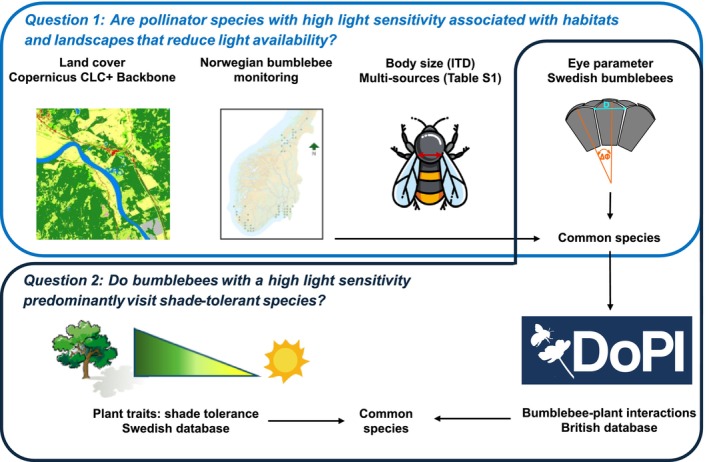
Diagram illustrating how the various data used in this study address our two research questions. For Question 1, we combined data from the Norwegian bumblebee monitoring program (Åström and Åström 2023), Copernicus CLC+ Backbone land‐cover data (European Union's Copernicus Land Monitoring Service information 2022), bumblebee body size (intertegular distance, ITD) compiled from multiple sources (Table S2), and eye parameter measurements from Tichit et al. (2024). The analysis considered only bumblebee species common to both the monitoring and eye parameter datasets. For Question 2, we focused on the same bumblebee species complemented with *Bombus subterraneus*. Bumblebee–plant interactions were retrieved from a British database (Balfour et al. 2022) and data on shade tolerance from a trait database (Tyler et al. 2021) (restricted to plants naturally growing in Sweden). Body size data were drawn from multiple sources to increase representativeness, while eye parameter measurements were taken from individuals captured in Sweden (i.e., at similar latitudes compared to Norway). The bumblebee–plant interactions were focused on bumblebee species present both in Norway and Britain, that is, with a wide distribution range, such as the plant species they interacted with—present both in Britain and Sweden—which has a similar latitudinal range and flora to Norway.

We used the Copernicus CLC+ Backbone (10 × 10 m, from 2018), which includes 11 basic land‐cover classes (European Union's Copernicus Land Monitoring Service information 2022) to estimate the forest cover (%) within a 1 km radius buffer around the centroid of each transect, as most bee species have a flight range below 1 km (Kendall et al. 2022) (Figure 2). Forest cover was calculated as the sum of the percentage cover of both coniferous (land‐cover class 2 “Woody—needle leaved trees”) and broadleaf (land‐cover class 3 “Woody—broadleaved deciduous trees”) forests using the exact_extract function in the *exactextract* package (Baston 2022). GIS analyses were performed in R v.4.3.0 (R Core Team 2023) with the packages *raster* (Hijmans 2023), *sf* (Pebesma and Bivand 2023), and *exactextractr* (Baston 2022).

### 2.3 Visual Trait Measurements

To investigate whether the visual abilities of bumblebee species might explain their relationships with habitat, landscape, and the plants they forage on, we used a visual trait known as the eye parameter. This is a measure that reflects the tradeoff between light sensitivity and visual resolution (Jander and Jander 2002) (Figure 1A). The eye parameter is also a practical trait to use because it is only weakly related to body size, with nonsignificant allometric scaling between the eye parameter and body size and correlations being more strongly driven by within, rather than between species differences (Tables S3 and S4 in Tichit et al. 2024). This study uses species averages from Tichit et al. (2024). The eye parameter was estimated from bumblebees sampled outside the monitoring range, with average measurements taken across one eye of each individual. It was calculated by multiplying the facet diameter (μm) by the average angle (in radians) between the centers of two adjacent facets. For investigating how the eye parameter interacts with habitat and landscape forest cover, values from workers for noncuckoo species and queens for cuckoo species were included, as these were the most commonly observed castes during monitoring (Table S1). Due to logistic and technical difficulties associated with measuring the eye parameter, only one or a few measures were obtained for each species (range: 1–7, mean: 1.7; details in Table S1). For six species with at least three individual measurements, repeatability analyses showed that interspecific differences explained 80% of the variance in eye parameter (Table S3 in Tichit et al. 2024). Eye parameter was not related to body size for the species/caste included in this study (linear mixed model: eye parameter ~ITD + (1|species)). Considering that Tichit et al. (2024) showed weak phylogenetic constraints on eye parameter variation, phylogeny was not included in the analyses. To relate bumblebee visual abilities to the light niche of the plants they forage on, we used the eye parameters of workers for bumblebee species (11 species, nine from the previous analyses and two less abundant species)—and the queen trait for the cuckoo species (three species, two from the previous analyses and one less abundant species) (Figure 2). In total, we included 15 species (Table S1).

### 2.4 Body Size—Intertegular Distance (ITD)

To control for potential body size effects on species responses across scales, ITD measures were compiled and averaged from several literature sources for workers of noncuckoo species and queens of cuckoo species (Table S2) (del Castillo and Fairbairn 2012; Kendall et al. 2019; Massa et al. 2024; Peat et al. 2005; Streinzer and Spaethe 2014; Tichit et al. 2024) (Figure 2). For the *B. terrestris* complex, values available for both *B. terrestris* and *B. lucorum* were averaged (Table S2). Body sizes of the individual used for eye parameter measurements were representative of the average size of their species as found in the literature (See Table S3).

### 2.5 Plant–Bumblebee Interactions and Plant Shade Tolerance

By testing the hypothesis that the eye parameter is related to the shade tolerance of the plant species bumblebees forage on, we aim to enhance our understanding of the behaviors—flying through and/or foraging—that are enabled by the visual abilities of different species in low‐light conditions. Data on bumblebee–plant interactions were obtained from the Database of Pollinator Interactions (DoPi) (Balfour et al. 2022), which focuses on pollinators in the United Kingdom, including 14 out of the 20 bumblebee species encountered in the Norwegian bumblebee monitoring data (Figure 2). *B. subterraneus* was also included as its eye parameter was available. We chose to focus on widely available data to include a broader range of plant species than would be possible using single studies. Data were downloaded for each bumblebee species separately. For the *B. terrestris* complex, selection was done through the option “*Bombus lucorum/terrestris*.” All plants with reported interactions in the DoPI database were included. To refine our search to Scandinavian plant taxa, we combined this data with a plant trait database compiled for Swedish plant species (Tyler et al. 2021) (Figure 2). We focused on the trait “light index,” a semiquantitative measure of the “optimal light/shade conditions of the species” from 1 (deep shade) to 7 (always full sun), based on expert knowledge and flora atlases.

The raw plant data from DoPi included 853 plant taxa (species and genus when reported as “spp.”). All unique bumblebee species–plant taxon interactions were included. For plant taxa reported at the genus level, average light index trait values across species of that genus were used. From these 853 taxa, 596 were initially found in the Swedish database. A manual search for taxonomic synonyms of the 257 missing taxa added an extra 19 taxa, resulting in the inclusion of 615 plant taxa in the analyses. Plant–bumblebee interactions related to the plant taxa absent of the Swedish plant trait database were removed. The numbers of unique bumblebee species–plant taxa associations are detailed in Table S1.

### 2.6 Statistical Analysis

All statistical analyses were conducted using R (R Core Team 2023). Generalized linear mixed models (GLMMs) were fitted with the *glmmTMB* package (Brooks et al. 2017). Residual diagnostics were checked with the *DHARMa* package (Hartig 2022). Post hoc contrast analyses were performed with the *emmeans* package (Lenth 2023). Visualizations were created using *ggplot2* (Wickham 2016), *sjPlot* (Lüdecke 2024), *cowplot* (Wilke 2025), and *ggstatsplot* (Patil 2021).

#### 2.6.1 Are Pollinator Species With High Light Sensitivity Associated With Habitats and Landscapes That Reduce Light Availability?

To test whether visual abilities influence how bumblebees interact with local habitat and landscape characteristics, we pooled community data per transect across 9 years of sampling to exclude interannual variation as the focus is on the spatial patterns and address zero inflation of the yearly data—resulting in 1081 transects. We transformed the data into “long format,” with each row representing the number of individuals observed for one bumblebees species on a particular transect, resulting in 12,972 observations (12 species × 1081 transects). We referred to the percentage of forest at the landscape scale as “forest cover” and the local habitat type of individual transects as “forest habitat” and “grassland habitat.” Two GLMMs were built to evaluate the effects of local and landscape characteristics simultaneously, as described in Table 1. The response variables for Models 1 and 2 were the probability of presence and the abundance of bumblebee species, respectively. A binomial distribution was chosen for Model 1, and a negative binomial distribution with a quadratic parameterization was chosen to address over‐dispersion for Model 2. Variation in sampling effort was accounted for by including logged sampling effort as a covariate in Model 1 (as offsets are not implemented for binomial models) and as an offset in Model 2. Both models included two crossed random effects: (i) sampling site identity to account for potential spatial autocorrelation, and (ii) species identity to account for species‐specific differences in occurrence rates and abundance. Geographical variation related to the three regions where bees were monitored was controlled by integrating latitude, longitude, and their interaction as fixed effects. Species body size was included in interaction with both environmental variables to account for its potential role in species responses to forest cover and habitat.

**TABLE 1:** Description of the four statistical models: Two generalized linear mixed models (Models 1 and 2), one linear mixed model (Model 3), and one linear model (Model 4).

Model (distribution)	Response variable	Formula	Random effect	Data sources
Model 1 (Binomial)	Species occurrence	Eye parameter * (habitat type + forest cover) + ITD * (habitat type + forest cover) + longitude * latitude + log(sampling effort)	+ (1|Site ID) + (1|species)	Norwegian bumblebee monitoring data Eye parameter from Tichit et al. (2024)
Model 2 (Negative binomial with quadratic parametrisation)	Species abundance	Eye parameter * (habitat type + forest cover) + ITD * (habitat type + forest cover) + longitude * latitude + offset (log (sampling effort))	+ (1|Site ID) + (1|species)	Norwegian bumblebee monitoring data Eye parameter from Tichit et al. (2024)
Model 3 (Gaussian)	CWM eye parameter	Habitat type + forest cover + latitude * longitude	+ (1|Site ID)	Norwegian bumblebee monitoring data Eye parameter from Tichit et al. (2024)
Model 4 (Gaussian)	Plant light index	Eye parameter		Plant‐bumblebee interaction from United Kingdom (Balfour et al. 2022) Eye parameter from Tichit et al. (2024) Plant light index from Swedish data base (Tyler et al. 2021)

*Note:* The response variables are: (i) species occurrence (binomial, 1/0), (ii) species abundance (integer), (iii) community‐weighted mean of the eye parameter (CWM, μm.rad), and (iv) the light index of foraged plant taxa (discrete, 1 to 7). The explanatory variables are: (i) eye parameter of each species (μm.rad), (ii) intertegular distance of each species (ITD, mm), (iii) habitat type (categorical, forest/grassland), (iv) forest cover in a 1 km radius (continuous, %), and (v) sampling effort is the number of times a transect was visited (integer). In Models 1–3, we fitted the identity of the 47 sites of sampling as a random effect to control for potential spatial autocorrelation between transects in Models 1–3. In Models 1 and 2, we also fitted the species ID as a random effect to account for species‐specific differences in occurrence rates and abundance.

For Models 1 and 2, post hoc contrast analyses on significant interaction terms were performed using the emtrends function of the *emmeans* package. For the interaction between the eye parameter (and ITD) and forest cover, we focused on the contrast between the slopes of three eye parameter (ITD) values: high (average + SD), intermediate (average), low (average—SD). For the interaction between the eye parameter and habitat, we compared the slopes estimates of trait value for the two habitat types. To account for the high abundance of the *B. terrestris* /*lucorum* group, we ran similar analysis excluding this species group. As the results were highly similar, we retained them in the analyses.

To test whether the observed patterns were consistent at the community level, we calculated the community‐weighted mean (CWM) of the eye parameter for the community averaged across the years at the transect level—that is, the averaged trait value weighted by the abundance of each bumblebee species:

$${\mathrm{CWM}}_{h}=\sum_{i=1}^{S}\;a_{\mathrm{ih}}\times t_{i}$$

where *a*_*ih*_ the abundance of species *i* across of the 10 years of sampling *h* and *t*_*i*_ the eye parameter value of the bumblebee species *i*. This was performed using the functcomp function of the *FD* package (Laliberté and Legendre 2010; Laliberté et al. 2014). Model 3 was built with the CWM of the eye parameter as the response variable and the same explanatory variables as Models 1 and 2, as described in Table 1, and sampling site ID as random effect.

#### 2.6.2 Plants and Bumblebees—Do Bumblebees With a High Light Sensitivity Predominantly Visit Shade‐Tolerant Species?

To investigate whether bumblebee species with higher light sensitivity forage mainly on shade‐tolerant plant species, we tested if the shade tolerance of the plant taxa visited by each bumblebee species is related to the light sensitivity of the bumblebees, as measured by the eye parameter. We built linear Model 4 with the light index of each plant taxon visited by individual bumblebee species as the response variable and the bumblebee species eye parameter as the explanatory variable. As each bumblebee species‐plant taxon was unique, we did not include a random effect.

#### 2.6.3 Potential Application in Species Distribution Modeling

To explore the potential of this study for species distribution modeling and its potential support for conservation management decisions given a wider availability of eye parameter measurements, we used Model 1 (Table 1) restricted to the geographical area around Oslo—thus excluding latitude and longitude. As the forest cover was measured in a 1 km radius around the centroid of the monitoring transects, we created a raster layer with the value of forest cover in this radius for each pixel using the following steps: (1) creation of a layer with only forest cover; (2) aggregation of the forest cover in 100‐by‐100 m pixels—to facilitate the computation, using the function aggregate of the terra package (Hijmans 2024); (3) calculation of forest cover within a 1 km radius around each pixel with the moving window function focalMat (Hijmans 2024). A raster stack was created, including this first raster layer, a layer for sampling effort, and a layer for the random effect. The predict function was used to create species occurrence distribution maps from the raster stack and Model 1. Two maps were created: one for a species with a high eye parameter value ( *B. pratorum* ) and one for a species with a low eye parameter value ( *B. wurflenii* ), which has similar ITD to create species‐specific maps while focusing on the effect of the eye parameter.

## 3 Results

### 3.1 Bumblebee Species With High Light Sensitivity Are Associated With Habitats That Have Reduced Light Availability

**FIGURE 3:**
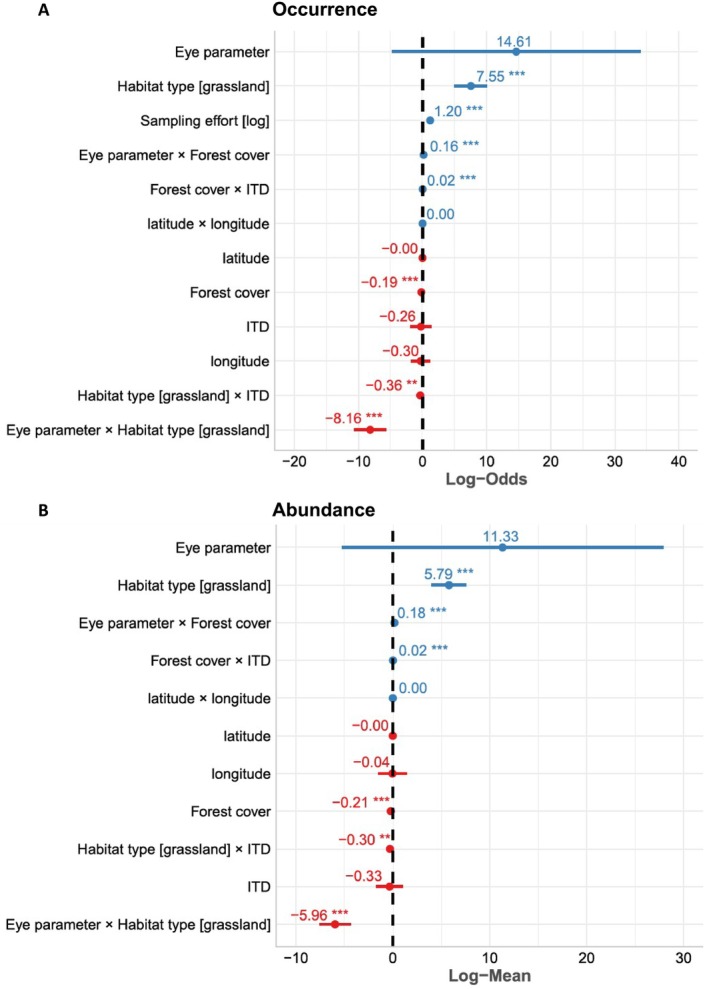
(A) Log‐odd ratios of binomial Model 1 (species occurrence) and (B) log‐mean generalized Poisson Model 2 (species abundance). In both models, random effects (sampling sites) were included and had a variance of 1.98 × 10^−4^ for Model 1 and 1.56 × 10^−2^ for Model 2. Blue indicates positive values, red indicates negative values. Bars indicate the 95% confidence interval. The vertical black dashed line is the 0 value. *** indicates *p* < 0.001. ** indicates 0.001 ≤ *p* < 0.01.

The occurrence and abundance of bumblebee species were higher in grassland than in forest habitats (occurrence: *Z* = 5.84, *p* < 0.001; abundance: *Z* = 6.37, *p* < 0.001) (Figure 3). Species such as *B. sylvarum* , *B. lapidarius* , *B. wurflenii* , and *B. terrestris* were more abundant in grasslands, while *B. jonellus* and *B. pratorum* were more abundant in forests. Both occurrence and abundance decreased with increasing forest cover (occurrence: *Z* = −7.65, *p* < 0.001; abundance: *Z* = −12.38, *p* < 0.001), as observed with species like *B. sylvarum* , *B. lapidarius* , and *B. terrestris* , in contrast to *B. jonellus* , *B. pratorum* , and *B. pascuorum* (Figure 3).

**FIGURE 4:**
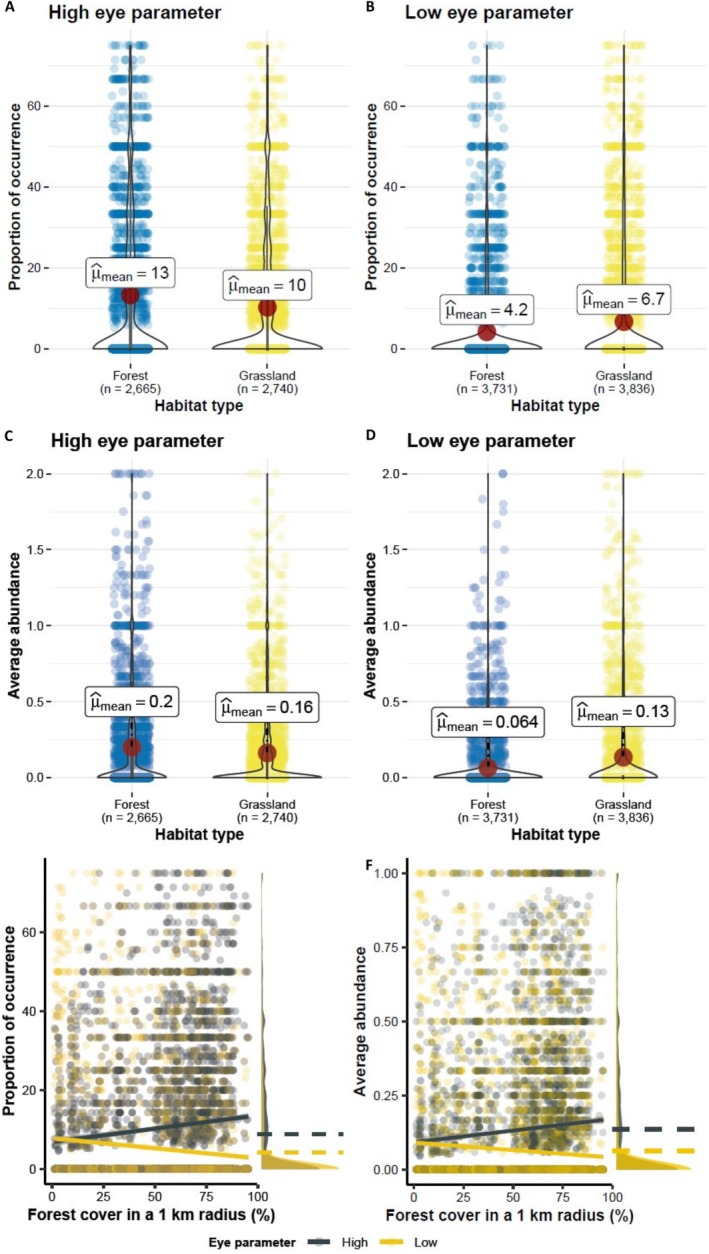
Post hoc contrast analyses of the modeling of bumblebee communities (Models 1 and 2). The interaction between the eye parameter of bumblebee species with the habitat type (forest, grassland) and (A, B) bumblebee occurrence—here measured as the proportion of sampling round where a species was detected on a transect, (C, D) bumblebee abundance—here measured as the average number of individuals per species for each sampling round on a transect; as well as for the interaction of the eye parameter with the forest cover in a 1 km radius of (E) bumblebee occurrence and (F) bumblebee abundance where the side panels display the distribution density of proportion of occurrence and average abundance for low and high parameter bumblebee species. In all panels, the “high eye parameter” included the bumblebee species with an eye parameter higher than the average across species while the “low eye parameter” included the bumblebee species with an eye parameter lower than the average across species. This division was made for illustration purposes. In Panels A–D, each dot stands for the bumblebee occurrence (A, B) and abundance (C, D) for each of the monitored transects. The blue color displays transects monitored in forests; the yellow color refers to transects monitored in open habitats. The violin plots the distribution of bumblebee occurrence (A, B) and abundance (C, D) and $\hat{\mu_{\text{mean}}}$ is the average value of each variable. In Panels E, F, each dot stands for the occurrence (E) and abundance (F) of bumblebees with high eye parameter (in gray) and low eye parameter (in yellow). The regression lines represent the response of bumblebee occurrence and abundance to forest cover. Despite proportion of occurrence being as high as 10% and average abundance as high as 10 individuals, we limited the axis range to a maximum of 10% of proportion of occurrence and an abundance of two individuals to exclude the outliers and display the relationships more clearly. In the side panels, the dotted lines represent the average value of occ occurrence (E) and abundance (F) of bumblebees with high eye parameter (in gray) and low eye parameter (in yellow).

Our hypothesis was evaluated by post hoc contrasts, showing consistent results for both occurrence and abundance. Bumblebees with higher eye parameters had higher occurrence and abundance in forest habitats compared to grassland habitats (occurrence: *Z* = 6.54, *p* < 0.001; abundance: *Z* = 7.27, *p* < 0.001), exemplified by *B. pratorum* and *B. pascuorum* (Figure 4A–D, Table 2).

**TABLE 2:** Post hoc contrast analyses of the interactions between eye parameter and forest cover (%), respectively, and the habitat type (grassland, forest) for both the occurrence and abundance models (Models 1 and 2).

Post hoc contrast analysis	Occurrence model emtrends slopes (95% CI)	Abundance model emtrends slopes (95% CI)
Interaction eye parameter and forest cover		
High eye parameter (mean + SD)	0.0110 [0.0068, 0.0151]	0.0057 [0.0027, 0.0088]
Medium eye parameter (mean)	0.0011 [−0.0024, 0.0046]	−0.0049 [−0.0076, −0.0021]
Low eye parameter (mean − SD)	−0.0103 [−0.0156, −0.0051]	−0.0173 [−0.0210, −0.0136]
Interaction ITD and forest cover		
High ITD (mean + SD)	0.0145 [0.0092, 0.0198]	0.0090 [0.0050, 0.0131]
Medium ITD (mean)	0.0004 [−0.0031, 0.0039]	−0.0057 [−0.0084, −0.0029]
Low ITD (mean − SD)	−0.0137 [−0.0188, −0.0086]	−0.0204 [−0.0243, −0.0165]
Interaction eye parameter and habitat type		
Forest	24.00 [4.85, 43.10]	21.40 [4.95, 37.90]
Grassland	15.80 [−3.29, 34.90]	15.50 [−1.00, 31.90]
Interaction ITD and habitat type		
Forest	0.74 [−0.84, 2.31]	0.71 [−0.65, 2.06]
Grassland	0.38 [−1.19, 1.95]	0.41 [−0.94, 1.77]

*Note:* Here we present the emtrend slopes together with their 95% confidence interval (CI). SD stands for standard deviation, ITD for inter‐tegular distance.

Analyses revealed that both the occurrence and abundance of species with high eye parameters increased along a forest cover gradient, as seen with *B. jonellus* and *B. pratorum* (occurrence: *Z* = −6.69, *p* < 0.001; abundance: *Z* = −11.37, *p* < 0.001) (Figure 4E,F, Table 2). In contrast, species with low eye parameters, such as *B. sylvarum* , showed decreased occurrence and abundance along the same gradient, while species with intermediate eye parameters, like *B. soroeensis* , showed no significant change with forest cover (95% CI overlapping with 0) (Table 2).

The occurrence and abundance of large bumblebee species, for example, *B. bohemicus* , increased with forest cover, whereas those of small species, such as *B. sylvarum* and *B. lapidarius* , decreased along the same gradient (occurrence: *Z* = 7.30, *p* < 0.001; abundance: *Z* = 10–12, *p* < 0.001) (Table 2). Large species had higher occurrence and abundance in forests than in grasslands (occurrence: *Z* = 3.02, *p* = 0.002; abundance: *Z* = 3.21, *p* = 0.001) (Table 2).

These patterns were consistent at the community level, with the CWM of the eye parameter being higher in forest compared to grassland habitats (*Z* = −4.61, *p* < 0.001) and increasing with forest cover (*Z* = 6.03, *p* < 0.001) (Figure S1).

### 3.2 Bumblebee Species With High Light Sensitivity Predominantly Visit Shade‐Tolerant Plant Species

The analysis revealed a significant relationship between the eye parameter and the light index of the plants each bumblebee species forages on. Specifically, as the eye parameter increased, the light index of the plants decreased (estimate: −1.02 ± 0.36, *Z* = −2.86, *p* = 0.004). This finding indicates that species with higher light sensitivity tend to visit plants with higher shade tolerance (Figure S2).

### 3.3 Potential Application in Species Distribution Modeling

**FIGURE 5:**
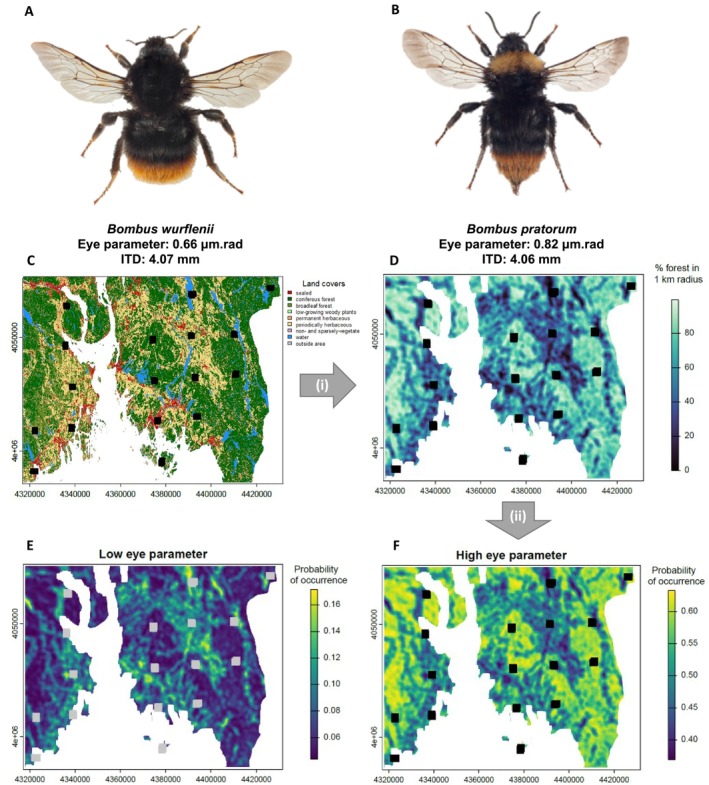
Creation of predictive maps for the occurrence of bumblebees based on their eye parameter value in function of forest cover with a focus on the sampling sites (black squares) located on the Oslo region with a focus on (A) a species with a low eye parameter is the worker *Bombus wurflenii* (eye parameter = 0.66 μm.rad) (CC BY 3.0, Arnstein Staverløkk) and (B) a species with a high eye parameter: The worker of *B. pratorum* has a high eye parameter (0.81 μm.rad (CC BY 3.0, Arnstein Staverløkk). (i) We use the forested areas (both coniferous and broadleaf forests, 10 × 10 m resolution)) of the land‐cover map (C) to create a raster at a coarser resolution (100 × 100 m) to calculate the proportion of forest cover in a 1 km radius around each pixel (D). (ii) This raster is used to spatially predict the probability of occurrence of bumblebees based on their eye parameter and body size (E, F).

Spatial distribution modeling of the presence of *B. pratorum* and *B. wurflenii* in the Oslo region, based on Model 1, resulted in contrasting distribution maps (Figure 5). *B. wurflenii* , characterized by low‐light resolution, was predominantly found in open landscapes (Figure 5C,E). In contrast, *B. pratorum* , known for high light sensitivity, was more commonly observed in forested landscapes (Figure 5D,F).

## 4 Discussion

In this study, we found that bumblebee species with the highest light sensitivity were more frequently observed in landscapes with increased forest cover, whereas species with the lowest light sensitivity were less commonly found in such landscapes. This pattern held true at the community level, with the community‐weighted mean of the eye parameter being higher in forest than in grassland and increasing in both habitats together with forest cover. Furthermore, bumblebee light sensitivity was correlated with the shade tolerance of the plants they forage on. Species with higher light sensitivity predominantly foraged on plants with greater shade tolerance. This finding supports our interpretation that bumblebee species navigate and forage in habitats consistent with their visual capabilities—according to their own limitations or adapting to the local environment. This suggests that their distribution may be influenced by landscape scales, impacting their presence in local open patches. These results offer new perspectives for both management and conservation efforts, as well as for species distribution modeling.

### 4.1 Bumblebee Vision and Habitat Use

At a broad geographic scale, climatic conditions filter bee species based on their temperature tolerance, which can be estimated through body size (Hoiss et al. 2012; Oyen et al. 2016). At a landscape scale, body size serves as a proxy for mobility, influencing foraging locations by determining flight range (Greenleaf et al. 2007; Kendall et al. 2022; Zurbuchen et al. 2010), and dispersal abilities (López‐Uribe et al. 2019). The eye parameter correlates well with bumblebee habitat selection, encompassing both navigation and foraging behaviors. Our findings suggest that bumblebee visual abilities may be involved in species distributions across spatial scales. These abilities not only help explain light microhabitat use in bilberry‐blooming forests (Bartholomée et al. 2023) but also how bumblebee species utilize their visual environment on larger scales. Our study extends the results of Tichit et al. (2024) which showed that grassland bumblebee communities exhibited higher light sensitivity with increased forest cover, by suggesting that the light sensitivity of both grassland and forest bumblebee communities increases with forest cover, generalizing the range of the habitats over which this trend can be observed. This enhances our understanding of bumblebee habitat use and the robustness of predictive models by extending the studied habitat range, with the eye parameter being the trait most responsive to changes in forest cover. The observed patterns might have developed over evolutionary times (Klausmeier et al. 2020), which can occur rapidly under environmental change and be driven by plant–pollinator interactions (Pontarp et al. 2024). Bumblebee sensory traits and habitat use may have co‐evolved such that (i) in ecological times, sensory traits affect habitat choice and habitat‐related fitness of bumblebee species, and (ii) in evolutionary times, for example, over longer periods of time, sensory traits could evolve in response to habitat affinity or habitat affinity could evolve in relation to sensory traits. The eco‐evolutionary process could be, for example, that the use of habitats with dimmer light conditions would select for higher light sensitivity, favoring the use of habitats with darker light conditions. This relationship may underlie bumblebee community assembly, as illustrated by the landscape‐scale effect of forest cover observed in this study. The potential coevolution is reinforced by the consistency between the dim light abilities of bumblebees and the shade tolerance of the plants they forage on.

Some functional traits—such as body size—are involved in different aspects of species' ecologies. Eye parameter appears to be related to species parasitism, with queens of parasitic species (subgenus *Psithyrus*) having a lower eye parameter—that is, higher visual resolution—than nonparasitic species (Tichit 2021). This might reflect a need for greater visual acuity to locate host nests. However, in this study, the eye parameter of the nonparasitic species was not related either to nest location (above‐ vs. below‐ground) or colony size (classified as small, medium, and large), reinforcing that the observed patterns are likely linked to species' visual abilities rather than to other correlated ecological traits. However, as we could not control for potential mechanisms covarying with light conditions, we cannot be sure that other evolutionary responses were not involved.

Our findings also indicate a potential role of body size in habitat association, with larger bee species being more prevalent in areas with higher forest cover. This observation suggests higher mobility for these species (Greenleaf et al. 2007; Kendall et al. 2022; Zurbuchen et al. 2010), enabling them to traverse greater distances across forested habitats in search of more flower‐rich open areas. Additionally, larger bees exhibit higher light sensitivity due to the allometric relationship between eye size and body size (Howland et al. 2004; Jander and Jander 2002), with larger eyes providing better light sensitivity (Liporoni et al. 2020; Somanathan et al. 2007). Similar patterns were observed in stingless bees, with larger bee species having a lower light intensity flight threshold compared to smaller bees, except for a tiny species with a high eye parameter, highlighting the role of the eye parameter in informing about a species' visual light sensitivity beyond the constraints of its size (Streinzer et al. 2016). From this perspective, future research should encompass a broader range of species and consider intraspecific variations in sensory traits, which may be adapted to local conditions but also allow colonies of social bees to exploit a wider range of environmental conditions. Since vision has been shown to influence microhabitat use in other taxa, such as butterflies (Wainwright et al. 2023), upscaling this analysis to habitat and landscape levels could help generalize these patterns to other insect taxa.

### 4.2 Bumblebee Vision and Forage Plants

Bumblebee visual abilities appear to be related to the light niche of the plants they forage on. Species with higher light sensitivity, and therefore a likely higher affinity for forest habitats or forested landscapes, tend to forage on plants with greater shade tolerance. This suggests that they are capable of navigating and foraging in dim environments, enhancing our understanding of the visually mediated association between bumblebee species and forest habitats. This pattern indicates that bumblebee visual traits may limit their ability to navigate through forests to commute between habitats, as well as their ability to forage on flowers in the forest understory. Species with a high eye parameter may have an advantage for both flying and foraging in low‐light conditions (Baird et al. 2015; Spaethe et al. 2001). While both landscape‐scale and local effects of habitat may influence fitness differentials, the results at local scales may also represent active habitat choice by bumblebees. However, how bumblebees and other pollinators interact with flowers is also influenced by abiotic factors (e.g., temperature, precipitation, pesticide exposure) and biotic factors (e.g., flower abundance, presence of conspecifics/other pollinators) (Cervantes‐Loreto et al. 2021), which were not accounted for in the data used in this analysis. By drawing consistent results with vision‐habitat analyses across two geographically distinct datasets, we reinforce our hypothesis regarding the central role that insect vision plays in shaping bumblebee habitat use. This approach also demonstrates the versatility of the relationships between visual traits and the bumblebee environment including habitat, landscapes, and foraged plants. This study lays the foundation for future studies investigating whether this pattern is consistent over larger geographical scales and in different species assemblages. Further investigations could explore if this pattern exists for other insect taxa. For example, future research could examine whether the light niches of butterfly host plants are consistent with the visual abilities of the species and whether this pattern can be observed at higher trophic levels.

### 4.3 Study Caveats

Although this study focuses on the role of visual abilities, this does not negate the influence of other functional traits on bumblebee distribution, such as diet breadth and nest location (Persson et al. 2015; Williams et al. 2010), as well as competition. This study relied on pooled or generalized data on a number of dimensions, and further studies could enhance these methods by using co‐occurring data in time and space on smaller scales (eye parameter measurements, land‐use data, plant–bumblebee interactions), as well as increase the sample size of bumblebees measured for visual traits. The small sample size may limit species representativeness for some traits; the greater variability in eye parameters between than within species (see Methods section) supports its use for generalizing the observed patterns, as highlighted by the repeatability measures in Tichit et al. (2024). We used mean trait values at the species level (Cantwell‐Jones et al. 2024), a widely accepted method in studies exploring species–environment interactions (e.g., Ekroos et al. 2013; Williams et al. 2010) and species distribution modeling (e.g., Pradervand et al. 2014; Suzuki‐Ohno et al. 2024; Torvanger et al. 2025). Plant–bumblebee interactions were not weighted by their frequency, which could skew analyses toward rarer interactions. Future studies could enhance these methods by combining bumblebee community monitoring with detailed flower inventories across habitats and landscapes to strengthen our understanding of functional responses.

## 5 Conclusions and Management Perspectives

This study investigated species–habitat associations across scales and examined the role of visual abilities in these patterns. Bumblebees navigate and forage in habitats and landscapes in relation to their light sensitivity. Species and communities with higher light sensitivity are associated with dim light habitats and landscapes and forage on plants that grow in dimmer light conditions. This indicates that how bees perceive and interact with their environment affects their distribution at multiple scales. We highlight the need to work toward developing more comprehensive datasets of eye parameter measurements to facilitate their use in ecological studies. We suggest thinking about conservation measures and management practices, particularly in forests, by taking into account habitat light intensity alongside the light sensitivity of bees and the plants they forage on. For instance, management actions in boreal forests could promote plants that support threatened pollinator species by fostering heterogeneous forests with diverse stand densities and age distributions (Gómez‐Martínez et al. 2020; Rhoades et al. 2018). Moreover, in addition to potential direct beneficial impacts on flower resources, forest canopies provide cool microclimates (De Frenne et al. 2019; Pincebourde and Woods 2020), which can buffer heatwaves and serve as refugia for insects, such as bumblebees, that are sensitive to extreme events (Martinet et al. 2021). Including sensory traits in species distribution modeling could generalize the observed patterns to bee species with unknown habitat preferences but known visual traits, improving our predictive abilities regarding bee responses to forest loss or habitat encroachment.

## Author Contributions

**Océane Bartholomée:** conceptualization (equal), formal analysis (lead), funding acquisition (supporting), methodology (lead), software (equal), visualization (lead), writing – original draft (equal), writing – review and editing (lead). **Pierre Tichit:** data curation (supporting), investigation (supporting), methodology (supporting), validation (equal), writing – review and editing (equal). **Jens Åström:** data curation (equal), formal analysis (supporting), investigation (equal), validation (equal), visualization (supporting), writing – review and editing (equal). **Henrik G. Smith:** formal analysis (supporting), writing – review and editing (equal). **Sandra Åström:** data curation (equal), investigation (lead), validation (equal), writing – review and editing (supporting). **Markus A. K. Sydenham:** conceptualization (equal), formal analysis (supporting), methodology (equal), software (equal), visualization (supporting), writing – review and editing (equal). **Emily Baird:** conceptualization (equal), funding acquisition (equal), investigation (supporting), supervision (equal), validation (supporting), writing – review and editing (equal).

## Conflicts of Interest

The authors declare no conflicts of interest.

## Supporting information


**TABLE S1:** Bumblebee species included in the analysis of the Question 1 (vision, habitat, and landscape) with their total abundance in the 10 years of monitoring data and the Question 2 (vision and floral resources light niche) with the number of plant taxa they were reported interacting with—and for which trait data was available. The shaded cells indicate species not included in the analysis.
**TABLE S2:** Traits for the bumblebee species included in the study: eye parameter (in μm.rad) and the inter‐tegular distance (ITD, in mm), together with the measurement sample size and the literature source where the data was taken from. For the ITD, we calculated an averaged value based weighted by the sample size of the different sources included in the calculation.
**TABLE S3:** Comparison of the intertegular (ITD) distance of the bumblebee individuals used for eye parameter measurements with the average ITD of their species as found in the literature. We calculated the weighted average for each species based on one to four sources (see Table S2) as well as the weighted standard deviation (SD) based on the average of each source, the overall mean and their sample size.
**FIGURE S1:** Community‐weighted mean of bumblebee eye parameter in forests and grasslands (Model 3) (*Z* = −4.61, *p* < 0.001) along a forest cover gradient (*Z* = 6.03, *p* < 0.001) (Model 3). The shaded area represents 95% confidence interval.
**FIGURE S2:** Average light index of plants foraged on by bumblebee species explained by their eye parameter (workers for all species except cuckoo queens) (Model 4). The shaded area represents the 95% confidence intervals. The figure represents the plant light index averaged for each bumblebee species when the model accounted for the individual light index of the plant taxon.

## Data Availability

The data and code will be made available on Dryad upon the article publication. For the review process, here is a peer‐review link: https://doi.org/10.5061/dryad.g4f4qrg1p.
