# Supplementary material for: Light Sensitive Bumblebee Species Are Associated With Forest Habitat and Forest‐Dominated Landscapes
Source: Ecol Evol. 2025 Oct 22;15(10):e72351. doi: 10.1002/ece3.72351 (PMC12545700; doi:10.1002/ece3.72351)
Supplement: Supplementary file 1 — TABLE S1: Bumblebee species included in the analysis of the Question 1 (vision, habitat, and landscape) with their total abundance in the 10 years of monitoring data and the Question 2 (vision and floral resources light niche) with the number of plant taxa they were reported interacting with—and for which trait data was available. The shaded cells indicate species not included in the analysis. TABLE S2: Traits for the bumblebee species included in the study: eye parameter (in μm.rad) and the inter‐tegular distance (ITD, in mm), together with the measurement sample size and the literature source where the data was taken from. For the ITD, we calculated an averaged value based weighted by the sample size of the different sources included in the calculation. TABLE S3: Comparison of the intertegular (ITD) distance of the bumblebee individuals used for eye parameter measurements with the average ITD of their species as found in the literature. We calculated the weighted average for each species based on one to four sources (see Table S2) as well as the weighted standard deviation (SD) based on the average of each source, the overall mean and their sample size. FIGURE S1: Community‐weighted mean of bumblebee eye parameter in forests and grasslands (Model 3) (Z = −4.61, p < 0.001) along a forest cover gradient (Z = 6.03, p < 0.001) (Model 3). The shaded area represents 95% confidence interval. FIGURE S2: Average light index of plants foraged on by bumblebee species explained by their eye parameter (workers for all species except cuckoo queens) (Model 4). The shaded area represents the 95% confidence intervals. The figure represents the plant light index averaged for each bumblebee species when the model accounted for the individual light index of the plant taxon. [file ECE3-15-e72351-s001.zip › ece372351-sup-0001-TableS1-S3-FigureS1-S2@SUPPLEMENTARY MATERIAL.docx]

# **Supplementary Information**

**Table S1:** Bumblebee species included in the analysis of the question 1 (vision, habitat and landscape) with their total abundance in the ten years of monitoring data and the question 2 (vision and floral resources light-niche) with the number of plant taxa they were reported interacting with – and for which trait data was available. The shaded cells indicate species not included in the analysis.

| **Bumblebee species** | **Parasitic species** | **Vision, habitat and landscape** | | **Vision and floral resources light-niche** | | **Sample size**  **eye parameter measure** |
| --- | --- | --- | --- | --- | --- | --- |
|  |  | **Included** | **Total abundance** | **Included** | **Reported number of interactions** |  |
| B. bohemicus | Yes | Yes | 190 | Yes | 41 | 1 |
| B. campestris | Yes | Yes | 33 | Yes | 44 | 1 |
| B. hortorum | No | Yes | 298 | Yes | 265 | 2 |
| B. hypnorum | No | Yes | 820 | Yes | 232 | 1 |
| B. jonellus | No | Yes | 177 | Yes | 58 | 1 |
| B. lapidarius | No | Yes | 1047 | Yes | 371 | 1 |
| B. monticola | No | No | 19 | Yes | 35 | 1 |
| B. muscorum | No | No | 4 | Yes | 102 | 1 |
| B. pascuorum | No | Yes | 3797 | Yes | 455 | 4 |
| B. pratorum | No | Yes | 2457 | Yes | 314 | 1 |
| B. rupestris | Yes | No | 3 | Yes | 44 | 2 |
| B. soroeensis | No | Yes | 30 | Yes | 19 | 1 |
| B. subterraneus | No | No | 0 | Yes | 3 | 1 |
| B. sylvarum | No | Yes | 77 | Yes | 37 | 1 |
| B. terrestris* | No | Yes | 4522 | Yes | 312 | 7 |
| B. wurfelini | No | Yes | 40 | No | 0 | 1 |

#### *B. terrestris includes here both B. lucorum and B. terrestris, as both species are highly difficult to distinguish in the field (Carolan et al. 2012)**.**

Carolan JC, Murray TE, Fitzpatrick Ú, Crossley J, Schmidt H, Cederberg B, McNally L, Paxton RJ, Williams PH, Brown MJF (2012) Colour Patterns Do Not Diagnose Species: Quantitative Evaluation of a DNA Barcoded Cryptic Bumblebee Complex. PLOS ONE 7:e29251. doi: 10.1371/journal.pone.0029251

**Table S2:** Bumblebee functional traits – detailed in a separate Excel file.

**Table S3:** Comparison of the inter-tegular (ITD) distance of the bumblebee individuals used for eye parameter measurements with the average ITD of their species as found in the literature. We calculated the weighted average for each species based on one to four sources (see Table S2) as well as the weighted standard deviation (SD) based on the average of each source, the overall mean and their sample size.

| **Bumblebees species** | **Caste included in study** | **Eye parameter (μm.rad) (sample size)** | **ITD Average weighted by the number of individuals from the literature**  **(mm)** | **Weighted Standard Deviation** | **Total sample size** | **Sources*** | **Average ITD**  **(Tichit et al., 2024)** | **Sample size**  **(Tichit et al., 2024)** | **SD**  **(Tichit et al., 2024)** | **Is ITD from the eye parameter individual in the literature average +/- SD** |
| --- | --- | --- | --- | --- | --- | --- | --- | --- | --- | --- |
| *B.bohemicus* | Queen  (cuckoo species) | 0.595 (1) | 6.40 |  | 1 | 1 | 6.40 | 1 |  | X |
| *B. campestris* | Queen  (cuckoo species) | 0.620 (1) | 6.00 |  | 1 | 1 | 6.00 | 1 |  | X |
| *B. hortorum* | Worker | 0.784 (2) | 4.56 | 0.39 | 193 | 2, 3, 4, 5 | 4.36 | 2 | 0.19 | Yes |
| *B. hypnorum* | Worker | 0.726 (1) | 3.69 | 0.17 | 26 | 3, 4 | 4.70 | 1 |  | No |
| *B. jonellus* | Worker | 0.755 (1) | 4.03 | 0.36 | 35 | 2, 3 | 3.70 | 1 |  | Yes |
| *B. lapidarius* | Worker | 0.695 (1) | 4.07 | 0.44 | 203 | 2, 3, 4, 6 | 4.96 | 1 |  | No |
| *B. monticola* | Worker | 0.667 (1) | 3.73 | 0.23 | 18 | 1, 6 | 4.64 | 1 |  | No |
| *B. muscorum* | Worker | 0.637 (1) | 4.87 |  | 507 | 2 | 4.50 | 1 |  | Close |
| *B. pascuorum* | Worker | 0.784 (4) | 3.72 | 0.04 | 102 | 2, 4, 6 | 3.97 | 4 | 0.19 | Yes |
| *B. pratorum* | Worker | 0.818 (1) | 4.06 | 0.31 | 230 | 2, 4 | 4.01 | 1 |  | Yes |
| *B. rupestris* | Queen  (cuckoo species) | 0.571 (2) | 6.49 | 0.58 | 3 | 1, 4 | 6.84 | 2 | 0.25 | Yes |
| *B. soroeensis* | Worker | 0.708 (1) | 3.91 | 0.24 | 64 | 2, 6, 7 | 3.80 | 1 |  | Yes |
| *B. subterraneus* | Worker | 0.614 (1) | 4.10 | 0.52 | 3 | 1, 3 | 4.70 | 1 |  | Close |
| *B. sylvarum* | Worker | 0.656 (1) | 4.11 | 0.21 | 62 | 2, 3, 4 | 4.15 | 1 |  | Yes |
| *B. terrestris*** | Worker | 0.671 (7) | 4.66 | 0.44 | 671 | 2, 4, 6 | 4.15 | 7 | 1.33 | Yes |
| *B. wurfelini* | Worker | 0.658 (1) | 4.07 | 0.09 | 81 | 3, 6, 7 | 4.70 | 1 |  | No |

*1. Tichit et al. (2024); 2. Peat, Tucker, and Goulson (2005); 3. del Castillo and Fairbairn (2012); 4. Kendall et al. (2019) ; 5. Massa, Hille Ris Lambers, and Richman (2024); 6. Massa et al. (2024); 7. Streinzer and Spaethe (2014)

** *B. terrestris* includes here both *B. lucorum* *and B. terrestris*, as both species are highly difficult to distinguish in the field (Carolan et al. 2012).


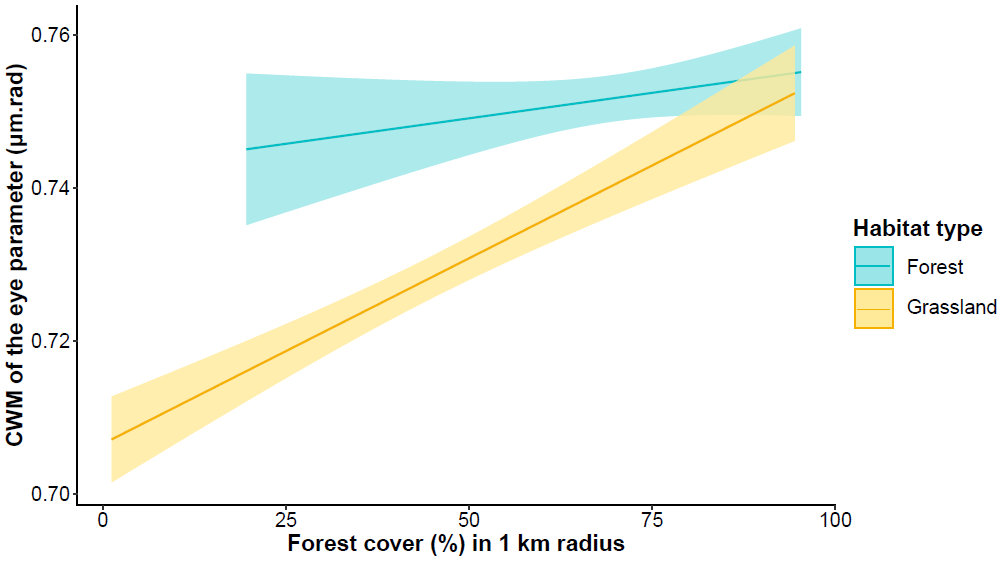


#### **Figure S1:** Community-weighted mean of bumblebee eye parameter in forests and grasslands (Model 3) (Z = -4.61, P < 0.001) along a forest cover gradient (Z = 6.03, P < 0.001) (Model 3). The shaded area represents 95% confidence interval.

**
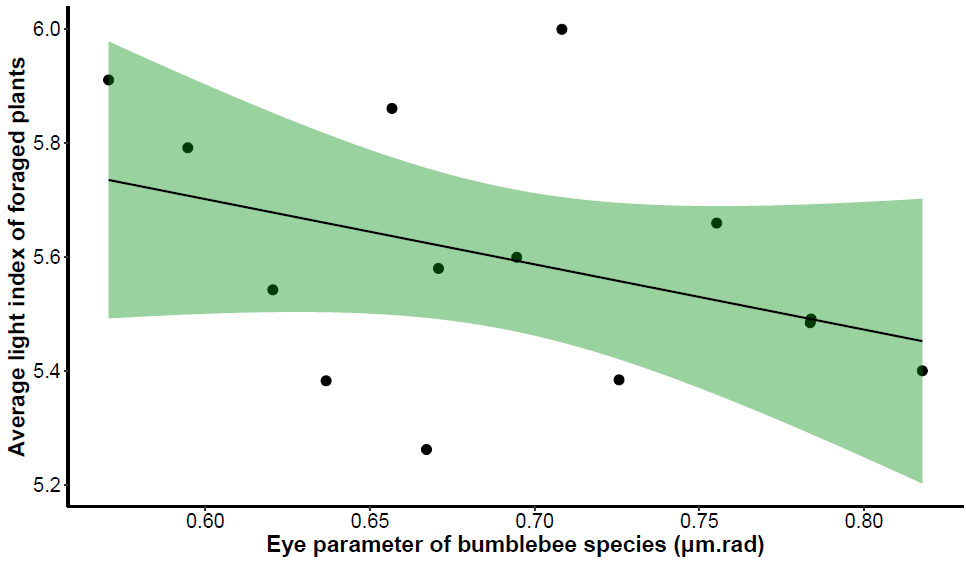
**

**Figure S2:** Average light index of plants foraged on by bumblebee species explained by their eye parameter (workers for all species except cuckoo queens) (Model 4). The shaded area represents the 95% confidence intervals. N.B.: The figure represents the plant light index averaged for each bumblebee species when the model accounted for the individual light index of the plant taxon.

## **Data availability statement**

The data and code will be made available on Dryad upon the article publication. For the review process here is a peer-review link: <http://datadryad.org/share/XtRb3iVCakQ6hb7c-gmWytKrosdF-vhaSy7_NZOIWHc>.
